# Supplementary material for: Depression and Functioning during the COVID-19 Pandemic among Adults across Tunisia
Source: Int J Environ Res Public Health. 2024 Oct 16;21(10):1363. doi: 10.3390/ijerph21101363 (PMC11506968; doi:10.3390/ijerph21101363)
Supplement: Supplementary file 1 [file ijerph-21-01363-s001.zip › ijerph-3209115-supplementary.pdf]

Table S1

Intercorrelations among variables

Scale	1	2	3	4	5	6	7	8	9	10	11
1. Age	--	-.011	.477**	-.077*	.135**	.238**	.170**	-.038	-.037	-.011	.174**
2. COVID-19	-.026	--	-.027	.021	.063*	.085**	.022	.075*	.048	.092**	.076*
3. Vaccinated	.470**	-.021	--	-.060	-.026	.071*	-.013	.034	.012	-.060	.048
4. Trauma	-.069*	.028	.015	--	.084**	.101**	.051	.013	-.064*	.275**	.164**
5. Family	.070*	-.041	-.028	.018	--	.339**	.482**	-.043	-.094**	.355**	.269**
6. Health Problems	.151**	.043	.050	.057	.299**	--	.373**	-.102**	-.099**	.376**	.370**
7. Financial Problems	.057	-.050	-.082**	.019	.496**	.300**	--	-.079*	-.085**	.345**	.237**
8. Friend support	-.007	.054	.040	-.047	-.108**	-.064*	-.157**	--	.185**	-.100**	-.111**
9. Family support	.072*	-.006	.034	-.054	-.048	-.005	-.058	.264**	--	-.163**	-.117**
10. Depression	-.078*	.003	-.069*	.118**	.401**	.326**	.391**	-.160**	-.151**	--	.530**
11. Impaired Functioning	.044	.018	.039	.087**	.296**	.316**	.268**	-.158**	-.133**	.523**	--

Note: The correlations in the upper half of the matrix are for females and the correlations on the bottom half of the matrix are for males. COVID-19 = confirmed diagnosis 0 = no, 1 = yes. Vaccinated 0 = not vaccinated against COVID-19, 1 = vaccinated. Trauma 0 = reported no history of trauma, 1 = *p < .05, **p < .01, ***p < .001.
